# A novel small molecule target in human airway smooth muscle for potential treatment of obstructive lung diseases: a staged high-throughput biophysical screening

**DOI:** 10.1186/1465-9921-12-8

**Published:** 2011-01-13

**Authors:** Steven S An, Peter S Askovich, Thomas I Zarembinski, Kwangmi Ahn, John M Peltier, Moritz von Rechenberg, Sudhir Sahasrabudhe, Jeffrey J Fredberg

**Affiliations:** 1 Division of Physiology, Department of Environmental Health Sciences, Johns Hopkins Bloomberg School of Public Health, Baltimore, MD 21205, USA; 2 Prolexys Pharmaceuticals, Inc., Salt Lake City, UT 84116, USA; 3 Division of Biostatistics, Department of Public Health Sciences, Penn State College of Medicine, Hershey, PA 17033, USA; 4 Program in Molecular and Integrative Physiological Sciences, Harvard School of Public Health, Boston, MA 02115, USA

## Abstract

**Background:**

A newly identified mechanism of smooth muscle relaxation is the interaction between the small heat shock protein 20 (HSP20) and 14-3-3 proteins. Focusing upon this class of interactions, we describe here a novel drug target screening approach for treating airflow obstruction in asthma.

**Methods:**

Using a high-throughput fluorescence polarization (FP) assay, we screened a library of compounds that could act as small molecule modulators of HSP20 signals. We then applied two quantitative, cell-based biophysical methods to assess the functional efficacy of these molecules and rank-ordered their abilities to relax isolated human airway smooth muscle (ASM). Scaling up to the level of an intact tissue, we confirmed in a concentration-responsive manner the potency of the cell-based hit compounds.

**Results:**

Among 58,019 compound tested, 268 compounds caused 20% or more reduction of the polarized emission in the FP assay. A small subset of these primary screen hits, belonging to two scaffolds, caused relaxation of isolated ASM cell *in vitro *and attenuated active force development of intact tissue *ex vivo*.

**Conclusions:**

This staged biophysical screening paradigm provides proof-of-principle for high-throughput and cost-effective discovery of new small molecule therapeutic agents for obstructive lung diseases.

## Background

For treatment of bronchospasm in asthma, a well known target is the β_2_-adrenergic receptor (β_2_-AR) on smooth muscle that wraps circumferentially around the conducting airways [1]. By triggering relaxation of this airway smooth muscle (ASM), the conventional inhaled β_2_-agonists alleviate constriction of the airway lumen driven by ASM contraction and thereby relieve airflow obstruction. However, not all asthmatic patients respond equally well to inhaled β_2_-agonists [2-4], and some even experience accelerated lung function decline [5,6]. The primary pathway by which β_2_-agonists modulate ASM contraction is through activation of adenylyl cyclase, resulting in accumulation of intracellular 3',5'-cyclic adenosine monophosphate (cAMP) and subsequent activation of cAMP-dependent protein kinase (PKA) [1,7]. PKA then mediates multiple downstream signals that culminate in ASM relaxation [7-9].

One of the major protein substrates for PKA is the small heat shock protein 20 (HSP20) [10-12], and phosphorylation of HSP20 is now linked to relaxation of both airway and vascular smooth muscle [10-15]. The mechanistic action of HSP20 phosphorylation is incompletely understood, however [11,16-18]. Recently, Dreiza and colleagues [19] have demonstrated that the phosphorylated form of HSP20 (pHSP20) interacts with 14-3-3 proteins, which are considered the "gatekeepers" of actin depolymerizing protein cofilin [20-22]. Hence, mounting evidence points to the molecular interaction between pHSP20 and a class of 14-3-3 proteins as a critical determinant of cofilin-mediated disruption of actin stress fibers and smooth muscle relaxation [15,19,23].

Here we focused on pHSP20 and 14-3-3 γ protein interactions as molecular targets. We designed a staged high-throughput screen in human ASM for the discovery of potential small molecule therapeutic agents against airflow obstruction in asthma. First, we screened a library of compounds that could act as small molecule modulators of pHSP20-14-3-3 γ protein interactions using a high-throughput fluorescence polarization (FP) assay. We then tested the effects of these small molecule analogs of pHSP20 on cell stiffness and cell traction force exercised by human ASM. At the level of a single ASM cell, we measured changes in cell stiffness using magnetic twisting cytometry (MTC) and changes in cell traction force using Fourier transform traction microscopy (FTTM). Finally, scaling up to the level of an intact tissue, we validated the potency of the cell-based hit compounds using experimental animals in *ex vivo *setting.

## Methods

### Materials

Bovine trachea were collected from a local slaughterhouse (Dale T Smith & Sons Inc., Draper, UT) and transported to the laboratory in cold (4°C) bicarbonate buffer containing 120 mM NaCl, 4.7 mM KCl, 1.0 mM MgSO_4_, 1.0 mM NaH_2_PO_4_, 10 mM glucose, 1.5 mM CaCl_2_, and 25 mM Na_2_HCO_3 _(pH 7.4). Tissue culture reagents were obtained from Sigma (St. Louis, MO) with the exception of Dulbecco's modified Eagles's medium (DMEM)-Ham's F-12 (1:1) which was purchased from GIBCO (Grand Island, NY). The synthetic arginine-glycine-aspartic acid (RGD) containing peptide was purchased from American Peptide Company (Sunnyvale, CA). Primary antibodies against HSP20, cofilin, phosphorylated cofilin and 14-3-3 γ proteins, as well as the appropriate secondary antibodies, were obtained from Millipore (Billerica, MA). Unless otherwise noted, all other reagents were obtained from Sigma. Acetylcholine, histamine, serotonin, isoproterenol, and N^6^,2'-O-dibutyryladenosine 3',5'-cyclic monophosphate (db-cAMP) were reconstituted in sterile distilled water, frozen in aliquots, and diluted appropriately in serum-free media on the day of use.

### Statement on animal welfare

Fischer 344 rat strains (male, 7-9 wk-old) were purchased from Harlan Sprague-Dawley, Inc. (Indianapolis, IN) and housed in a conventional animal facility at Harvard School of Public Health (Boston, MA). All experimental protocols conducted on animals were performed in accordance with the standards established by the US Animal Welfare Acts, as well as the Policy and Procedures Manual of the Harvard University School of Public Health Animal Care and Use Committee.

### Isometric force measurements

As described previously by us and others [14,24], bovine tracheal strips and rat tracheal rings (i.e. transverse rings, 1.0 mm in width) were prepared and mounted in organ bath containing a bicarbonate buffer. Tissue strips/rings were tied with surgical silk and attached at one end to a force transducer (Kent Scientific, Litchfield, CT). The other end of tissue strips/rings were connected to a length manipulator. Tissue strips/rings were progressively stretched to a total force of ~10 g and then released to a passive force of ~0.5 g. Subsequently, the isometric force in response to a contracting agonist acetylcholine was determined until a consistent maximal force was produced. Here we used sub-maximally activated tissue strips/rings (~80% of the maximal contraction with 3 μM acetylcholine) and used 5% w/v cyclodextrin as a vehicle for the delivery of compounds. For each pre-contracted tissue, compounds were added directly to the organ bath. To ensure the viability of the tissue, the isometric force in response to 110 mM KCl (with equimolar replacement of NaCl in bicarbonate buffer) was measured after each experiment.

### Cell isolation and culture

Smooth muscle (i.e. vascular and airway) cells were isolated from either the aorta or the trachealis of the highly inbred Fischer 344 rat strains (male, 7-9 wk-old) as described previously [15,25]. Human ASM cells were isolated, characterized and provided by Dr. Reynold A. Panettieri, Jr. (University of Pennsylvania). Cells were grown until confluence at 37°C in humidified air containing 5% CO_2 _and passaged with 0.25% trypsin-0.02% EDTA solution every 10-14 days. ASM cells in culture were elongated and spindle shaped, grew with the typical hill-and-valley appearance, and showed positive staining for the smooth muscle-specific protein α-actin and calponin. In the present study, we used cells in passages 3-7. Unless otherwise specified, serum-deprived post-confluent cells were plated at 30,000 cells/cm^2 ^on plastic wells (96-well Removawell, Immunlon II: Dynetech) previously coated with type I collagen (Vitrogen 100; Cohesion, Palo Alto, CA) at 500 ng/cm^2^. Cells were maintained in serum-free media for 24 h at 37°C in humidified air containing 5% CO_2_. These conditions have been optimized for seeding cultured cells on collagen matrix and for assessing their mechanical properties [25-31].

### Magnetic twisting cytometry (MTC)

Stiffness of the adherent ASM cell was measured as described by us in detail elsewhere [25,29,32]. In brief, an RGD-coated ferrimagnetic microbead (4.5 μm in diameter) bound to the surface of the cell was magnetized horizontally and then twisted in a vertically aligned homogenous magnetic field that varied sinusoidally in time. The sinusoidal twisting magnetic field causes both a rotation and a pivoting displacement of the bead: as the bead moves, the cell develops internal stresses which in turn resist bead motions [29]. Lateral bead displacements in response to the resulting oscillatory torque were detected optically (with a spatial resolution of ~5 nm), and the ratio of specific torque to bead displacements was computed and expressed here as the cell stiffness in units of Pascal per nm (Pa/nm).

For each individual cell, stiffness was measured continuously for the duration of 600 s (Additional file 1, Figure S1): baseline stiffness was measured for the first 0-60 s and stiffness changes in response to compounds were measured up to the indicated time (60-600 s). In general, changes in cell stiffness approached a steady-state level within 600 s. In the present study, we reported this steady-state cell stiffness (540-600 s) upon treatment with various compounds. Moreover, to adjust for cell-to-cell and day-to-day variability in baseline stiffness, we normalized stiffness changes to respective baseline stiffness of an individual ASM cell.

### Fourier transform traction microscopy (FTTM)

The contractile stress arising at the interface between each adherent cell and its substrate was measured with traction microscopy [25,27]. Cells were plated sparsely on elastic gel blocks (Young's modulus of 8 kPa with a Poisson's ratio of 0.48), and allowed to adhere and stabilize for 24 h. For each adherent cell, the traction field was computed using Fourier transform traction cytometry as described previously [33,34]. The computed traction field was used to obtain the net contractile moment, which is a scalar measure of the cell's contractile strength [33]. The net contractile moment is expressed in units of pico-Newton meters (pNm).

### Protein expression/phosphorylation detection

The expression of HSP20, cofilin, and phosphorylated cofilin was detected as previously described [19,35]. For each well of confluent ASM cells (on 6-well plates), total cell protein was quantified by the Bradford method (using Bio-Rad dye reagent, Richmond, CA), and equal amounts of protein were resolved by SDS-PAGE and transferred to nitrocellulose membrane. Membranes were blocked and then probed with primary antibodies to HSP20, cofilin or phosphorylated cofilin. Immunoreactive proteins were detected with appropriate secondary antibodies and visualized by light emission on film with enhanced chemiluminescent substrate (Cell Signaling, Danvers, MA).

### Surface plasmon resonance (SPR) assay

All SPR experiments were performed on a BIAcore 3000 instrument. Phosphorylated HSP20 (pHSP20) peptide was immobilized to one flow cell of a CM5 chip (BIAcore) via a standard amino coupling procedure. The other three flow cells contained immobilized unphosphorylated HSP20 peptide (HSP20), a phosphorylated peptide containing a scrambled sequence of the pHSP20 peptide, and an empty surface blocked with ethanolamine, respectively. The 5 different 14-3-3 isoforms (β, ζ, η, ε and ϒ), expressed and purified from *E. coli *(described in detail below), were injected separately at equal concentrations in HBS (HEPES Buffered Saline, pH 7.4) with a flow rate of 20 μl/min across the pHSP20 and control surfaces. The dissociation was monitored for ca. 12 min in a HBS flow. Between injections, the surfaces were regenerated with a 30s pulse of 10 mM NaOH. The signal obtained from the HSP20 peptide surface were subtracted from that of the pHSP20 peptide surface.

### Fluorescence polarization (FP) assay

The 58,019 structurally diverse chemical compounds were obtained from ChemBridge (San Diego, CA) and ChemDiv (San Diego, CA). 8-mer peptides containing the recognition motif for 14-3-3 proteins were synthesized and purified via HPLC to > 95% purity, and their size confirmed by mass spectrometry (BioSynthesis, Inc., Lewisville, TX). The sequences of 8-mer peptides used were: 1) fluorophore-pHSP20 (6-FAM-WLRRApSAP); 2) positive control (WLRRApSAP); and 3) negative control (WLRRASAP).

The 247-amino acid 14-3-3γ coding region was cloned as a fusion with an N-terminal GST-His tag using the vector pDEST15 (Life Technologies) with expression under the control of the T7 promoter. BL21 (DE3) competent cells were transformed with pDEST15- GST-His14-3-3γ. Transformed bacteria were inoculated in 100 mL of LB media containing ampicillin at 10 μg/mL and grown overnight at 37°C. The overnight culture was diluted 1:50 in 4 L of fresh LB with the same concentration of antibiotic as described above. These cells were allowed to grow at 37°C for approximately 2-3 h, until the optical density at 600 nm reached 0.4 to 0.8. Induction was started by addition of IPTG at a final concentration of 0.1 mM, followed by incubation at 30°C for 5 h. Cells were harvested by centrifuge at 5000 rpm for 30 min. The cell pellet was resuspended, sonicated and centrifuged, and the soluble protein was subjected to two-step GST-His tag affinity purification according to manufacturer's instructions (Sigma-Aldrich Inc., St. Louis, MO; Qiagen Inc., Valencia, CA). Fractions containing GST-His-14-3-3γ (determined through SDS-PAGE) were pooled, and the protein concentration measured using the Bradford protein assay (Bio-rad, Hercules, CA). GST-His-14-3-3γ purity was assessed by SDS-PAGE and Coomassie Blue staining. This method was also used to prepare the other 14-3-3 isoforms used in the Surface Plasmon Resonance (SPR) experiments.

For the FP assay, we used 384-well microplates (low-volume, flat-bottom, black plates; Greiner-Bio-One North America Inc., Monroe, NC). First, GST-His-14-3-3γ and FAM-pHSP20 were added to the wells at final concentrations of 1 μM and 2 nM, respectively, in a final reaction buffer of 1X HBS-EP (0.01 M HEPES, pH 7.4, 0.15 M NaCl, 0.005% (v/v) polysorbate 20, 3 mM EDTA, 10 mM MgCl_2_). Compounds or negative/positive controls were then added at final concentrations of 10 μM and 1 μM, respectively. After 4 h incubation at room temperature, the FP was read using Perkin-Elmer Fusion Universal Microplate Analyzer (Perkin-Elmer, Shelton, CT) using 485 nm excitation (light-emitting diode) and 515 nm emission (20 nm bandwidth) settings. Compounds eliciting a variation of FP greater than 20% were flagged as optically active. After initial screening, flagged compounds were verified for inhibition in a concentration-responsive manner in order to establish their IC_50 _concentrations. All FP reactions were assayed in triplicate for each compound.

### Statistical analysis

For the comparisons among treatments, we used two sample t-test, the Analysis of Variance (ANOVA) with adjusting for multiple comparisons by applying the Tukey's method, or the Wilcoxon test depending on the distribution of data. To satisfy the distributional assumptions associated with ANOVA, cell stiffness data were converted to log scale prior to analyses. All analyses were performed in SAS V.9.1, and the 2-sided P-values less than 0.05 were considered significant.

## Results and Discussion

### Targeting HSP20 signals in the end-effector of airway constriction

**Figure 1:**
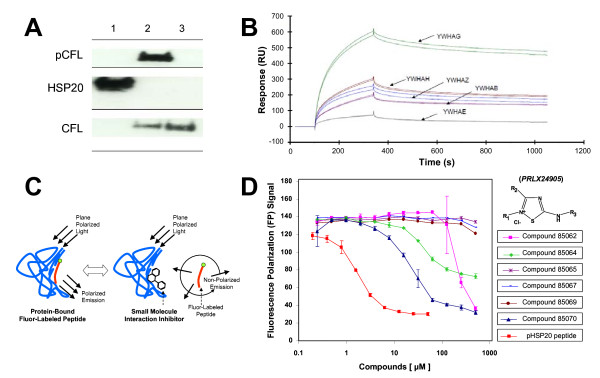
**Targeting pHSP20-14-3-3 protein interactions**. A. A representative Western blot (n = 3 separate experiments) using antibodies to HSP20 (lane 1), phosphorylated cofilin (lane 2), and cofilin (lane 3). B. A representative SPR-based evaluation of HSP20 binding to a class of 14-3-3 proteins. Synthesized peptides containing a partial sequence of phosphorylated HSP20 were immobilized via amine-coupling to a BIAcore chip, and GST-HIS-14-3-3 isoforms (YWHAG, γ; YWHAH, η; YWHAZ, ζ; YWHAB, β; and YWHAE, ε) were injected at 20 μg/ml. Experiments were conducted in triplicate. C. A schematic drawing of the principle behind the fluorescence polarization (FP) assay. FP signals of a flourophore is defined here as, FP = (V-H)/(V+H); where V is the vertical component and H is the horizontal component of the emitted light when excited by vertical plane polarized light. D. Changes in FP signals in response to a number of compounds belonging to the *PRLX24905 *scaffold (USA Patent & Trademark, Publication 20090136561: "Non-peptidyl agents with pHSP20-like activity, and uses thereof"). Data are presented as mean ± SE (n = 3 separate experiments).

Under basal conditions, human ASM cells expressed HSP20 and the actin-depolymerizing protein cofilin (Figure 1A), the latter of which was predominantly in its inactive phosphorylated form as reported earlier [12]. Phosphorylated cofilin is bound to 14-3-3 proteins [20-22] and, in human ASM, PKA-activated phosphorylation of HSP20 is associated with dephosphorylation of cofilin and subsequent loss of actin stress fibers [12]. Dreiza and colleagues [19] have demonstrated that phosphopeptide analogs of HSP20 (pHSP20) co-precipitate with a class of 14-3-3 proteins and, moreover, competitively inhibit the binding of phosphorylated cofilin to 14-3-3 proteins. Using SPR-based evaluation of protein interactions, we found that pHSP20 exhibited the highest binding affinity for the γ isoform of 14-3-3 proteins (Figure 1B). Hence, we focused on pHSP20-14-3-3 γ protein interactions in human ASM as a potential molecular target against excessive constriction of the airways in asthma.

### Screening small molecule modulators of pHSP20-14-3-3 γ protein interactions

Using a high-throughput *in vitro *FP assay, we screened a library of compounds that could act as small molecule modulators of HSP20 signals (Figure 1C). To this end, we employed a fluorophore-conjugated 8-mer peptide fragment of pHSP20 (6-FAM-WLRRApSAP) containing the recognition motif for 14-3-3 proteins; compared with the full-length pHSP20, this peptide fragment has a higher binding affinity for 14-3-3 γ proteins [19]. Among 58,019 compounds tested, 268 compounds caused 20% or more reduction of the polarized emission in FP assay (data not shown). Using the FP assay, therefore, we were able to quickly screen compounds that could modulate molecular interactions between pHSP20 and 14-3-3 γ proteins and find a number of promising scaffolds that could act as small molecule analogs of pHSP20. Here we limited our observations to a number of these tested scaffolds (both positive and negative).

Compounds belonging to one of the scaffolds (i.e. *PRLX24905*) showed a range of modulation of pHSP20-14-3-3 γ protein interactions in the FP assay (Figure 1D). For example, compounds *85065 *and *85067 *caused no reduction of the polarized emission, whereas compound *85070 *induced maximal reduction with an IC_50 _of approximately 50 μM. These compounds, together with structurally related scaffolds readily available from the supplier's catalogue, were re-ordered and re-tested for activity in a concentration-response manner. From these primary screen hits, we selected seven scaffolds and assessed their functional effects on cell stiffness and cell traction force exercised by human ASM. As previously demonstrated by us elsewhere [27], ASM cells maintain relatively high basal tone in culture that is attributable in large part to the dynamic interactions between actin and myosin. Unless otherwise noted, we assessed the effects of compounds on their abilities to decrease cell stiffness and cell traction force in the absence of contracting agonists.

### Testing functional efficacy of small molecule analogs of pHSP20

**Figure 2:**
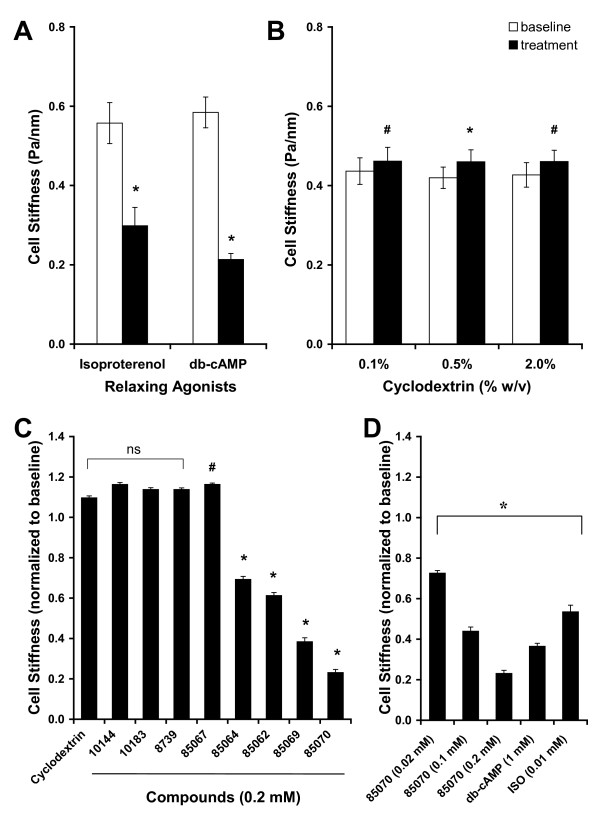
**Testing functional efficacy of small molecules with magnetic twisting cytometry**. A and B. The steady-state, stiffness prior to (baseline, open bars) and after the respective cell treatment (closed bars). Human ASM cells were treated for 10 min with (A) relaxing agonists (10 μM isoproterenol or 1 mM db-cAMP) and (B) buffer blank (0.1%, 0.5% or 2% w/v cyclodextrin). Stiffness is expressed as Pascal per nm (Pa/nm). Data are presented by geometric means, and error bars indicate standard error (SE); * indicates P < 0.001 and # indicates P < 0.05 from respective baseline stiffness (n = 152 to 442 cells). C and D. Stiffness responses of human ASM cells. Human ASM cells were (C) treated with vehicle control (0.5% w/v cyclodextrin) or a number of small molecules (200 μM) belonging to the *PRLX24905 *scaffold and (D) treated with an increasing concentration of compound *85070*. For comparison, stiffness responses to relaxing agonists (10 μM isoproterenol or 1 mM db-cAMP) are shown. Stiffness responses are normalized to respective baseline stiffness of an individual ASM cell. Data are presented by geometric means ± SE (n = 314 to 1024 cells); * indicates P < 0.001 and # indicates P < 0.05 from vehicle control.

At the level of a single ASM cell, we measured temporal changes in cell stiffness using MTC (Additional file 1, Figure S1). Over the course of 10 min, human ASM cells treated with either the β_2_-agonist isoproterenol or the cell-permeable cAMP analog dibutyryl-cAMP (db-cAMP) showed marked decreases in cell stiffness (Figure 2A). Cells treated with a buffer blank (0.1%, 0.5% or 2.0% w/v cyclodextrin) exhibited statistically significant increases in cell stiffness; however, the increases were less than 10% from the respective baseline stiffness. There were no statistical differences in the stiffness among cells treated with different cyclodextrin concentrations (Figure 2B). In this study, we chose 0.5% w/v cyclodextrin as a vehicle for the delivery of small molecules.

Among the seven scaffolds which showed activity in the FP assay as small molecule analogs of pHSP20, only a small subset of compounds belonging to two scaffolds caused appreciable decreases in cell stiffness. For instance, human ASM cells treated for 10 min with compounds belonging to the *PRLX24905 *scaffold exhibited a range of stiffness responses (Figure 2C). Compared to cells treated with vehicle control (0.5% w/v cyclodextrin), there were no statistical differences in stiffness responses of cells treated with compounds *10144*, *10183*, and *8739*. On the other hand, cells treated with compound *85067 *showed increases (P < 0.05) whereas cells treated with compounds *85064, 85062, 85069 *and *85070 *showed progressive decreases in cell stiffness (P < 0.001). Most strikingly, however, compound *85070 *that caused the greatest reduction of the polarized emission in the FP assay induced maximal decreases in cell stiffness (Figure 2C). Compound *85070 *also caused concentration-dependent decreases in cell stiffness (Figure 2D). Although the rate of decreases in cell stiffness by compound *85070 *was slower than that by β_2_-agonist isoproterenol (Additional file 1, Figure S1), we found that compound *85070 *was more efficacious in decreasing the stiffness of the human ASM cell than that by either the β_2_-agonist isoproterenol or the cell-permeable analog of cAMP (db-cAMP).

**Figure 3:**
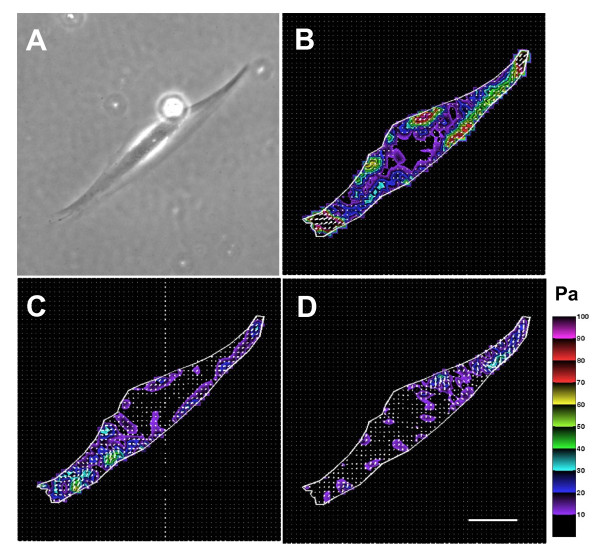
**Spatiotemporal changes in cell traction forces**. Phase contrast (A) and traction field images (B, 0 min; C, 5 min; D, 10 min) of a single human ASM cell treated with compound *85070*. Colors show the magnitude of the tractions in Pascal (Pa), and arrows show the direction and relative magnitude of the tractions. Scale bar, 50 μm. This is a representative of cells (n = 4) treated with 200 μM compound *85070*.

Consistent with stiffness responses, human ASM cells treated with compound *85070 *exhibited both spatial and temporal decreases in contractile force as measured by traction microscopy (Figure 3). Over the course of 10 min, compound *85070 *significantly inhibited the ability of an individual human ASM cell to generate contractile force. For example, the net contractile moment, which is a scalar measure of cell's contractile strength [33], decreased from 36.2 pNm (median, n = 4) at time zero to 7.9 pNm at 5 min and 3.1 pNm by 10 min upon incubation with compound *85070 *(P < 0.01; Wilcoxon test). Such decreases were significant (P < 0.05; Wilcoxon Test) when compared with time-matched cells treated with vehicle control (0.5% w/v cyclodextrin). For cells treated with vehicle control, there were no statistically significant changes in the net contractile moment (38.4 pNm at time zero to 40.3 pNm at 5 min and 36.9 pNm by 10 min; median, n = 3).

### Validation of the cell-based hit compounds

Scaling up to the level of an intact tissue, we tested the potency of these cell-based hit compounds in *ex vivo *setting. For these studies, we used trachealis rings prepared from inherently hyper-responsive Fischer rats [25,36,37]. For each trachealis ring, we measured responses of the intact tissue to a contracting agonist acetylcholine in a concentration-responsive manner. We limited our observations to compound *85070 *belonging to the *PRLX24905 *scaffold.

**Figure 4:**
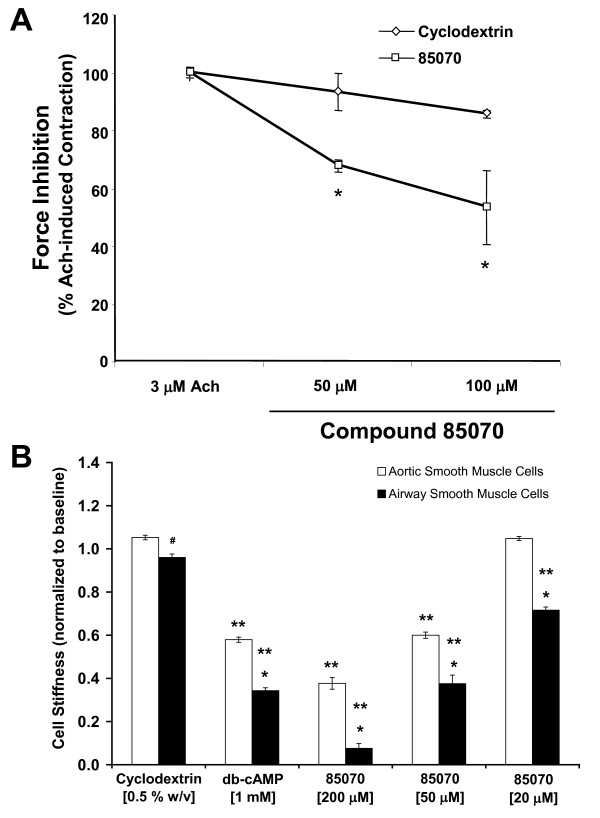
**Validation of the cell-based hit compounds**. **A**. Force inhibition of pre-contracted ASM tissues from inherently hyper-responsive Fischer rats. Tracheal rings were first contracted for 10 min with acetylcholine (3 μM) and subsequently treated with increasing concentrations of compound *85070*. For control, we used 5% w/v cyclodextrin. Data are presented as mean ± SE (n = 4 separate experiments). **B**. Stiffness responses of smooth muscle cells isolated from aorta and trachealis of the inherently hyper-responsive Fischer rats. Cells were treated with vehicle control (0.5% w/v cyclodextrin), dibutyryl-cAMP (1 mM), or compound *85070 *(20 μM, 50 μM or 200 μM). Stiffness changes are normalized to respective baseline stiffness of an individual cell. Data are presented by geometric means ± SE (n = 127 to 505 cells). For each treatment, * indicates P < 0.001 and # indicates P < 0.05 between the cell types. For each cell type, ** indicates P < 0.001 when compared with respective vehicle control.

For each tissue pre-contracted with a sub-maximal concentration of acetylcholine, compound *85070 *decreased the force generating capacity of rat trachealis (Figure 4A). Compound *85070 *also decreased the force generating capacity of muscle strips prepared from bovine trachealis (data not shown). Furthermore, as measured by MTC, compound *85070 *decreased the stiffness of ASM cells isolated from the trachealis of inherently hyper-responsive Fischer rats (Figure 4B). Such decreases in cell stiffness were concentration dependent and, when compared with cells isolated from the respective rat aorta (i.e. vascular smooth muscle), cells isolated from the trachealis showed greater decreases. Compound *85070 *also decreased the stiffness of serotonin-stimulated rat ASM cells, as well as histamine-stimulated human ASM cells (data not shown).

## Conclusions

To accelerate discovery, screening, testing and validation of new drug targets, here we have used a staged strategy that begins with a chemiproteomics-based approach [38] and progresses through quantitative biophysical assays at the levels of the isolated cell and then the intact tissue [25,32]. It remains unclear if the same cost-effective synergies of this staged approach might be applicable in the discovery of drug targets for other common diseases that involve changes in cell biophysical properties, including vasospasm, hypertension, heart failure, and cancer. As proof-of-principle, here we limited attention to the interaction of pHSP20 with 14-3-3 γ proteins, screened a library of 58,019 compounds, and discovered novel small molecule analogs of pHSP20 that might provide a therapeutic regime for obstructive lung diseases. At this time, we do not know whether these functional effects of small molecule analogs of pHSP20 are due to their direct actions of regulating actin filament dynamics [16,18], or indirect actions of displacing cofilin alone (Additional file 1, Figure S2) [19,20,22] or other regulatory protein kinases/phosphatases that interact with 14-3-3 proteins [21]. These mechanisms of actions are currently under investigation.

## List of abbreviations

ASM: airway smooth muscle; HSP20: heat shock protein 20; FP: fluorescence polarization; SPR: surface plasmon resonance; MTC: magnetic twisting cytometry; β_2_-AR: β_2_-adrenergic receptor; cAMP: 3',5'-cyclic adenosine monophosphate; PKA: cAMP-dependent protein kinase; db-cAMP: N^6^,2'-O-dibutyryladenosine 3',5'-cyclic monophosphate.

## Competing interests

SS, PSA, TIZ, JMP, and MR are former employees of Prolexys Pharmaceuticals Inc., and were compensated by the company at the time this work was performed. These employees have no financial arrangements with Prolexys at the present time. JJF and SSA received a consulting fee from Prolexys Pharmaceutical, Inc. At the present time, JJF and SSA have no financial relationship with Prolexys Pharmaceuticals. A part of this work (NON-PEPTIDYL AGENTS WITH pHSP20-LIKE ACTIVITY, AND USES THEREOF) has been applied for U.S. patent. There are no other competing interests or conflicts of interest.

## Authors' contributions

JJF, SS, and SSA conceived the high-throughput biophysical screening project. SSA, PSA, and JMP designed and implemented experimental protocols. JMP, TIZ, and MR conducted the FP assay. PSA, TIZ, and MR performed isometric force measurements of experimental animal models in *ex vivo *settings. TIZ and MR conducted pull-down assay and protein detection analysis. SSA isolated and cultured smooth muscle cells, and designed and performed all single-cell biophysical measurements. KA performed statistical analysis; KA and SSA analyzed the data. JJF and SS oversaw the project. SSA wrote the paper. All authors read and approved the final manuscript.

## Supplementary Material

Additional File 1 **Figures S1 and S2.** Figure S1: Temporal changes in cell stiffness as measured by magnetic twisting cytometry. Function efficacy of small molecules on stiffness of ASM at the level of a single living cell. Figure S2: Modulation of pCofilin-14-3-3 protein interactions. A potential mechanism of action of small molecules on relaxing ASM. Click here for file
