# Supplementary material for: A novel small molecule target in human airway smooth muscle for potential treatment of obstructive lung diseases: a staged high-throughput biophysical screening
Source: Respir Res. 2011 Jan 13;12(1):8. doi: 10.1186/1465-9921-12-8 (PMC3034681; doi:10.1186/1465-9921-12-8)
Supplement: Additional File 1 — Figures S1 and S2. Figure S1: Temporal changes in cell stiffness as measured by magnetic twisting cytometry. Function efficacy of small molecules on stiffness of ASM at the level of a single living cell. Figure S2: Modulation of pCofilin-14-3-3 protein interactions. A potential mechanism of action of small molecules on relaxing ASM. [file 1465-9921-12-8-S1.DOC]

**A novel small molecule target in human airway smooth muscle for potential treatment of obstructive lung diseases: a staged high-throughput biophysical screening**

Steven S An^1^ *, Peter S Askovich^2^ *, Thomas I Zarembinski^2^, Kwangmi Ahn^3^, John M Peltier^2^, Moritz von Rechenberg^2^, Sudhir Sahasrabudhe^2^, and Jeffrey J Fredberg^4^

Additional File 1:

**Figure S1: Temporal changes in cell stiffness as measured by magnetic twisting cytometry.** For each individual human ASM cell, stiffness was measured continuously for the duration of 600 s: baseline stiffness was measured for the first 0-60 s and stiffness changes were measured up to the indicated time (60-600 s) in response to media control, isoproterenol (10 μM), dibutyryl-cAMP (1 mM), vehicle control (0.5 % w/v cyclodextrin), compound *24069,* or compound *85070*. For changes in cell stiffness, the comparisons were tested using linear mixed-effects model by taking into account the repeated measurements after adjusting for baseline stiffness. Data are presented by geometric means, and error bars indicate standard error (SE) (n = 152 to 606 cells).

**Figure S2: Modulation of pCofilin-14-3-3 protein interactions.** Treated and untreated tissues (obtained from cow trachea and coronary artery) were solubilized in LDS buffer and equal volumes were loaded on an SDS-PAGE and blotted. Anti-phospho-cofilin (Anti-pCFL) antibody was used in a 1:1000 dilution and anti-GAPDH in 1:2000. Both were developed using a polyclonal donkey anti-rabbit antibody at 1:5000 dilution. Compared with untreated tissues, cow trachealis treated with either isoproterenol (ISO) or compound *86374* (a second working scaffold) showed increases of pCFL. The increases of pCFL are also visible in cow coronary artery by sodium nitroprusside (SNP) or compound *86374*. There were no changes of pCFL after treatment with contracting agonists acetylcholine (ACH) or serotonin (5HT), however. These findings suggest that, like pHSP20 peptide, the effectively screened compounds in FP and cell-based assays modulate pCFL-14-3-3 protein interactions, leading to ASM relaxation.
